# Convergence of lateral dynamic measurements in the plasma membrane of live cells from single particle tracking and STED-FCS

**DOI:** 10.1088/1361-6463/aa519e

**Published:** 2017-01-13

**Authors:** B Christoffer Lagerholm, Débora M Andrade, Mathias P Clausen, Christian Eggeling

**Affiliations:** 1 Wolfson Imaging Centre Oxford, Weatherall Institute of Molecular Medicine, University of Oxford, Headley Way, Oxford OX3 9DS, UK; 2 Centre for Neural Circuits and Behaviour, University of Oxford, Mansfield Road, Oxford OX1 3SR, UK; 3 MEMPHYS—Center for Biomembrane Physics, University of Southern Denmark, Campusvej 55, Odense M DK-5230, Denmark; 4 MRC Human Immunology Unit, Weatherall Institute of Molecular Medicine, University of Oxford, Headley Way, Oxford OX3 9DS, UK; christoffer.lagerholm@imm.ox.ac.uk

**Keywords:** diffusion, fluorescence correlation spectroscopy, cortical actin cytoskeleton, transient confinement

## Abstract

Fluorescence correlation spectroscopy (FCS) in combination with the super-resolution imaging method STED (STED-FCS), and single-particle tracking (SPT) are able to directly probe the lateral dynamics of lipids and proteins in the plasma membrane of live cells at spatial scales much below the diffraction limit of conventional microscopy. However, a major disparity in interpretation of data from SPT and STED-FCS remains, namely the proposed existence of a very fast (unhindered) lateral diffusion coefficient, ⩾5 *µ*m^2^ s^−1^, in the plasma membrane of live cells at very short length scales, ≈⩽ 100 nm, and time scales, ≈1–10 ms. This fast diffusion coefficient has been advocated in several high-speed SPT studies, for lipids and membrane proteins alike, but the equivalent has not been detected in STED-FCS measurements. Resolving this ambiguity is important because the assessment of membrane dynamics currently relies heavily on SPT for the determination of heterogeneous diffusion. A possible systematic error in this approach would thus have vast implications in this field. To address this, we have re-visited the analysis procedure for SPT data with an emphasis on the measurement errors and the effect that these errors have on the measurement outputs. We subsequently demonstrate that STED-FCS and SPT data, following careful consideration of the experimental errors of the SPT data, converge to a common interpretation which for the case of a diffusing phospholipid analogue in the plasma membrane of live mouse embryo fibroblasts results in an unhindered, intra-compartment, diffusion coefficient of  ≈0.7–1.0 *µ*m^2^ s^−1^, and a compartment size of about 100–150 nm.

## 1. Introduction

The relevant spatio-temporal scales for the fine structure of the mammalian plasma membrane are thought to be in the range of a few tens of nanometers to micrometers, and of tens of microseconds to seconds [1–5]. This is a combined scale range that few experimental approaches can access simultaneously. Electron microscopy, for example, has superior sub-nanometer image resolution combined with a capability of imaging specimen areas of tens to hundreds of micrometers but only at fixed time points and with very limited specific molecular staining options. In contrast, spectroscopy techniques, e.g. electron spin resonance (ESR), and nuclear magnetic resonance (NMR) are able to quantify short-range lateral motion of lipids at length-scales of a few nanometers at millisecond time-scales but cannot be extended to longer spatio-temporal scales. Finally, there exist a range of optical microscopy techniques e.g. fluorescence recovery after photo-bleaching (FRAP; also known as fluorescence photo-bleaching recovery (FPR)), various fluorescence correlation spectroscopy (FCS) techniques including scanning FCS (sFCS) and image correlation spectroscopy (ICS) methods (with variants such as STICS, RICS or iMSD), and single particle tracking (SPT) and related single molecule tracking (SMT) techniques. Of these techniques, only SPT and the combination of FCS techniques with the super-resolution imaging method STED are able to directly investigate lateral dynamics at diffraction-unlimited spatial scales ranging from tens to either hundreds of nanometers (FCS) or micrometers (SPT) while simultaneously accessing time-scales ranging from a few (STED-FCS) or tens (sSTED-FCS; SPT) of microseconds to seconds. But the experimental results from these different techniques have thus far not resulted in a consensus view for the organizational and functional properties of the plasma membrane.

There is in particular a need to resolve the disparity that was introduced with the interpretation that the lateral mobility of even phospholipids in the outer leaflet of the cellular plasma membrane of live cells is very strongly constrained by a mechanism that is dependent on the integrity of the cortical actin cytoskeleton [6, 7]. This interpretation, which originated from the implementation of high-speed SPT with a sampling frequency of 40–50 kHz, specifically concluded that a DOPE phospholipid analogue, that had been labelled with a colloidal gold particle and imaged at 37 °C, was transiently confined in the plasma membrane in compartments with a diameter of  ≈30–230 nm for a residence time of  ≈1–17 ms [6, 7]. The short-range, intra-compartment diffusion coefficient (*D*_*μ*_), defined as the free diffusion coefficient from a least-square unweighted analysis of the mean squared displacement (MSD) curves of exclusively sampling points 2–4, corresponding to a time regime of about 50–100 *µ*s, was found to be  ≈5– 8 *µ*m^2^ s^−1^ [6, 7]. This is a magnitude that is equivalent to diffusion coefficients that have only otherwise been observed for phospholipids in free-standing artificial membranes at much lower membrane protein densities than in cellular plasma membranes [8, 9]. In contrast, the long-term, inter-compartment, diffusion coefficient (*D*_MACRO_), defined by MSD analysis for a time window of 3 s, was found to be  ≈0.2 *µ*m^2^ s^−1^ for colloidal gold particle labelled DOPE and  ≈0.4 *µ*m^2^ s^−1^ for a fluorescent (Cy3) DOPE analogue. A direct measure of the confinement strength in these experiments is the ratio *S*_Conf_  =  *D*_*μ*_/*D*_MACRO_. The confinement strength for a colloidal gold labelled phospholipid analogue in the plasma membrane is thus  ≈25. Complementary studies using the same approach and by the same laboratory also showed that the intra-compartment diffusion was on the same scale for a range of membrane proteins (e.g. transferrin receptor, G-protein coupled receptors, and MHC class I) as for the phospholipid analogue [6, 7, 10, 11].

These results have been interpreted to originate from that the escape probability of a molecule that impacts a compartment boundary is very low such that the molecule will only very rarely be able to ‘hop’ to an adjacent compartment. This is referred to as the ‘the anchored-transmembrane protein picket fence model’ for which the compartment boundaries are hypothesized to be composed of a dense linear array of transmembrane proteins that are directly anchored to the sub-membranous F-actin cortex [6, 12]. Validation of these SPT inferred F-actin constrained compartments has been provided by direct visualization of the cortical actin network by optical tweezers [13] and electron tomography [14] but the presence of the proposed dense linear array of membrane protein pickets has not yet been detected. Alternatively, it has been proposed that a fast short-range but a slower long-range diffusion coefficient could also be the result of specific and non-specific lipid/protein interactions [15, 16] as has also been demonstrated by e.g. Monte Carlo simulations [17, 18]. Additionally, it has also been suggested that the observed lateral dynamics data could simply be caused by the inherent 3D topology of the plasma membrane which is generally neglected in the analysis of SPT data [19, 20]. But regardless of mechanism, the observed confinement as discussed above signifies very strong confinement, principally as a result of the very fast short-range diffusion coefficients.

The high-speed SPT results discussed above further remain controversial because the very fast diffusion coefficients has thus far only been observed in experiments at sampling frequencies of 40–50 kHz and by using large, 40 nm diameter, colloidal gold particles to label biomolecules of interest [6, 7, 10, 11, 21]. For example, a study by Wieser *et al* using either fluorescent dye (Alexa 647) labelled intact antibodies or antibody fragments to track CD59, a GPI-anchored protein, in T24 cells at 37 °C with a sampling time of 0.5 ms concluded that the data was best described as free diffusion with *D* (i.e. *D*  =  *D*_*μ*_  =  *D*_MACRO_)  ≈  0.3–0.5 *µ*m^2^ s^−1^, when labelling was with a minimally invasive Fab fragment, albeit with the reservation that weak transient confinement with a confinement strength *S*_Conf_  <  1.5 could not be ruled out [22]. In contrast, our SPT measurements using streptavidin conjugated quantum dots (sAv-QDs) at 1.8 kHz of a phospholipid (DPPE), a lipid-anchored protein (CD59), and a transmembrane receptor (EGFR) at room temperature (RT) in mouse embryo fibroblasts (MEFs) suggested that the most prevalent mode of diffusion of all three molecules was transient confinement (≈60–70% of trajectories) where other trajectories were best described as either free diffusion (≈5–10%) or confined diffusion (≈25–30%) [23]. The average analysis results for the three types of molecules, for the trajectories that were best described as transient confinement, were *L*  ≈  100–150 nm, *D*_*μ*_  ≈  0.3–0.6 *µ*m^2^ s^−1^, *D*_MACRO_  ≈  0.04–0.08 *µ*m^2^ s^−1^, and *S*_Conf_  ≈  8 [23]. Our recent STED-FCS data using a fluorescent dye (Atto647N) labelled phospholipid DPPE analogue at RT was also consistent with the concept that the confinement strength of the cortical actin cytoskeleton is sufficiently strong to constrain the lateral diffusion of a phospholipid analogue in compartments with a diameter of  ≈80 nm in NRK cells and  ≈150 nm in MEFs [24]. The short-range, unhindered, diffusion coefficient (*D*_*μ*_), defined in this case as the apparent diffusion coefficient at the smallest observation spot with a radius of  ≈40 nm was  ≈0.6 *µ*m^2^ s^−1^ in NRK and  ≈0.8 *µ*m^2^ s^−1^ in MEFs while the long-range, inter-compartment diffusion coefficient (*D*_MACRO_), defined as the apparent diffusion coefficient measured for the largest observation spot with a radius of  ≈250 nm was only two fold lower. The confinement strength in this study of *S*_Conf_  ≈  2 was thus also significantly weaker than that observed by high-speed SPT with colloidal gold particles as discussed above. This weaker confinement is further consistent with a recent iMSD study which found that GFP tagged transferrin receptor (GFP-TfR) in CHO cells at 37 °C is transiently confined for a short timescale (125 *µ*s–10 ms) to compartments of  ≈140 nm with a short-range diffusion coefficient of  ≈0.7 *µ*m^2^ s^−1^, a long-range diffusion coefficient of  ≈0.2 *µ*m^2^, and thus a confinement strength, *S*_Conf_  =  3.5 [5].

There are many possible reasons for the above disparities. First, it has been repeatedly argued that the reason that the very fast short-range diffusion coefficient has only been seen by using one specific approach for data acquisition and analysis is that no other method has the required combination of spatio-temporal resolution [2, 12, 25]. This is plausible and has further been validated by Monte Carlo simulations [26]. Yet recent methodological advances with in particular STED-FCS [24, 27, 28] and iMSD [5] have pushed the spatio-temporal resolution of these techniques to be very similar to that of high-speed SPT with colloidal gold particles. For example, STED-FCS with a spatial resolution of  ≈20 nm and a time-resolution of  ≈20 *µ*s (as determined by the measured transit time of a labelled phospholipid through an observation spot of a radius of  ≈20 nm in a model membrane) has been reported [29]. Further, even reported diffusion coefficients of phospholipids in cell blebs, presumably lacking cytoskeletal interactions such that it would be expected that diffusion measurements are independent of the spatio-temporal sampling, are much greater (~8–10 *µ*m^2^ s^−1^) as determined by high-speed SPT [6] than comparable measurements by e.g. FRAP (~1.2 *µ*m^2^ s^−1^) [30]. There have also been numerous concerns raised about the impact of the large colloidal gold particles which by necessity have been used in high-speed SPT [22, 31, 32]. It is furthermore intuitively unlikely that lipids should diffuse at the same coefficient in free standing membranes (lipid vesicles) as within sub-compartments of the plasma membrane, considering the potential steric effects caused by protein crowding on lipid diffusion in the plasma membrane as well as the potential friction effects induced by the glycocalyx on the apical plasma membrane of cells; many effects of which have been verified in model membranes [8, 9, 31]. An alternative cause of the disparity between high-speed SPT and STED-FCS measurements could thus also be that it originates from differences in data acquisition and analysis of the methods themselves, possibly as a consequence of that the measurement errors in each method are distinctly different.

In an attempt to resolve the above discussed disparity, we here present a review on the techniques of SPT and STED-FCS as these methods are presently unique in their ability to directly sample lateral membrane motion down to spatial and temporal scales that are adequate for the assessment of the plasma membrane nano-organization. We emphasize in particular the differences in data sampling, data analysis, and data interpretation. To demonstrate the effects of these differences, we present a data analysis example where we directly compare SPT [23] and STED-FCS data [24] of the lateral dynamics of a DPPE phospholipid analogue in the plasma membrane of live MEFs at RT. This demonstration shows that experimental diffusion measurements of a phospholipid analogue by STED-FCS and SPT converges to a consensus qualitative and quantitative result if we also incorporate the recent understanding of the influence of localization noise and camera blur in the SPT analysis [33–35].

## 2. Results and discussion

### 2.1. Single particle tracking

#### 2.1.1. Brief description of technique.

SPT is a camera based imaging technique in which time-lapse image sequences of sparsely labeled single molecules are acquired at fixed lag times, *t*_lag_, resulting in a sampling frequency of 1/*t*_lag_ [12, 31, 36–40]. Information about the motion of single molecules is subsequently obtained by image analysis whereby the typical first step is to determine the centroid position of each single molecule in each diffraction limited still frame of the image sequence. A pre-requisite for successful analysis in this case is that the density of labeling is sufficiently low such that all single particles are separated at least (and preferably by much more) than the distance given by the Rayleigh criterion, *d*  =  0.61*λ*/(NA), where *λ* is the wavelength of the fluorescence emission and NA is the numerical aperture of the objective [37, 39, 41–43]. The centroid of single molecules using this approach can typically be determined with a spatial precision of ~10–40 nm dependent on the signal to noise ratio, the image acquisition time, and the diffusion rate of the observed molecule [44]. The second step in this analysis is to link centroid positions of specific single molecules in time in order to form a series of single molecule trajectories. By further analysis of these trajectories, it is possible to distinguish between different types of motion, e.g. free Brownian diffusion, confined diffusion, and combinations thereof, and to assign quantitative values for the diffusion coefficient, and possible confinement sizes and confinement times [23, 39, 45]. This is most typically done by calculating the mean square displacement (MSD), either independently for each detected single molecule trajectory, or in the case where the single molecules trajectories are very short as an average for all detected single molecule trajectories [37, 39, 46]. Alternatively, it is also possible to obtain information about the single molecule motion either by analysis of the probability distribution of the squared displacements [39, 47], or by use of maximum likelihood estimator [33, 48, 49] or covariance-based estimator approaches [50]. It has also recently been shown that it is possible to generate MSD curves, by the so-called iMSD approach, directly from single particle image data by use of image correlation spectroscopy methods [5, 51]. Because this approach does not require that the centroids positions are first determined, the requirement that the density of the labeling is very low is much less stringent [5].

#### 2.1.2. Development of technique.

Quantitative SPT measurements are based on the theoretical formulation by Einstein in 1905 which established the relationship between the MSD, time *t*, and the diffusion coefficient *D* in one dimension

1 \begin{eqnarray*}\text{MS}{{\text{D}}_{\text{theory}}}(t)\,=\,\langle {{\left(x(t)-{{x}_{0}}\right)}^{2}}\rangle ={\int}_{-\infty}^{\infty}{{x}^{2}}N\left[0,2Dt\right]\,\text{d}x=\,2Dt\,\end{eqnarray*}

where *x*(*t*) denotes the position of a molecule at time *t*, *x*_0_ is the position of a molecule at time zero and *N*[0,2 *Dt*] is the normal distribution with zero mean and variance of 2*Dt* ([52]; also available in translation [53]). This derivation can be extended to *d* spatial dimensions as MSD_theory_(*t*)  =  2*dDt*. This theoretical framework was experimentally verified by Perrin and colleagues [54, 55] by observation of the Brownian motion of a dilute suspension of 1 *µ*m diameter gamboge particles by use of an ultra-microscope and a camera lucida with sampling intervals, *t*_lag_, of 30 s thus corresponding to a data sampling frequency, *f*_aq_  =  1/*t*_lag_  =  1/30 s  =  33.3 mHz. The work by Perrin *et al* whose main purpose was to obtain a mean estimate for Avogadro’s constant of 6.9  ×  10^23^, is considered by many as the first direct proof of the atomic theory [55, 56].

More recently, non-functionalized particles were used to investigate the retrograde motion of micron-sized particles on the dorsal lamella of migrating fibroblasts with a sampling time of 30 s [57] and later for a similar study that also analysed the Brownian motion aspects of the observed motion with a sampling time of 10 s [58]. This was followed by the first application of SPT for investigating the lateral diffusion of a specific membrane protein complex, the low-density lipoprotein (LDL)-receptor complexes in the plasma membrane of human fibroblasts by use of DiI labelled LDL, thus also being the first example of using fluorescence microscopy for SPT [59]. Later studies introduced the use of the much smaller highly scattering, 40 nm diameter, colloidal gold particles to track protein movement [60]. Initially, this was accomplished using charged gold particles in combination with bright-field microscopy, and later by use of antibody functionalized gold particles in combination with differential interference contrast microscopy [61]. The use of colloidal gold particles in combination with increasingly sensitive cameras enabled a dramatic acceleration of the sampling frequency; initially to 30 Hz, and more recently to rates up to 50 kHz [11]. The spatial and temporal resolutions of SPT are currently further pushed by the interferometric scattering (iSCAT) scheme [62, 63]. Further work has also shown applications of SPT for investigating the lateral motion of lipids and proteins in the plasma membrane that were labeled with smaller probes including single fluorescent dyes (with a sampling frequency of about 60−200 Hz [47, 64] and more recently at 2 kHz [65]), fluorescent proteins (at rates up to 200 Hz [66]), and fluorescent quantum dots (QDs) (initially at rates of about 13 Hz [67] and more recently at rates of 1.8 kHz [23]).

**Figure 1.**
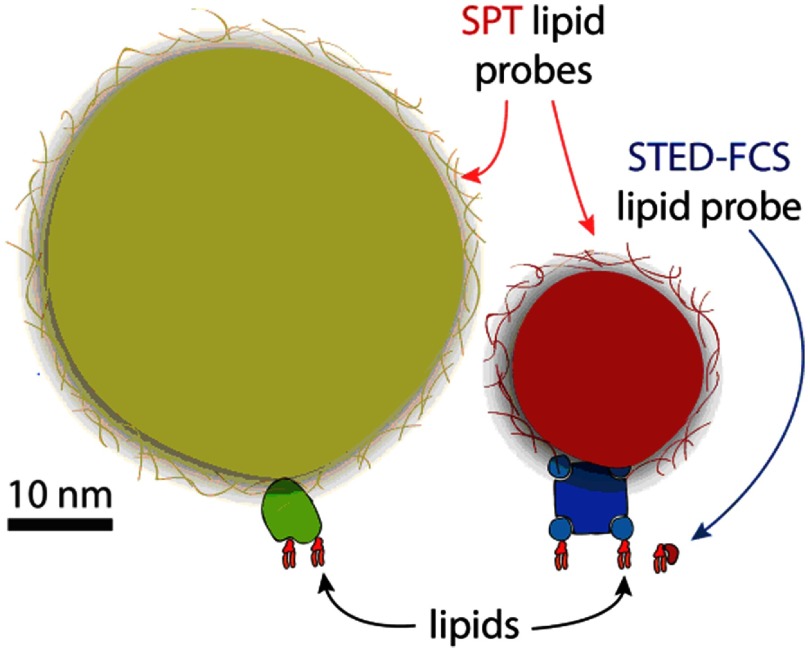
Comparison between lipid probes used in SPT and STED-FCS. Gold particle (≈40 nm in diameter) linked to lipid by Fab antibody and QD (≈20 nm in diameter) linked to lipid via streptavidin, as often used in SPT. Both probes are functionalized via polymer coating, which further enhances their effective size. Possible oligomerization induced by SPT probes is illustrated for the QD. STED-FCS lipid probe stands for a fluorescent lipid analogue (≈1 nm in diameter).

The properties of the probe particle have been shown to be very important for SPT [6, 51, 68]. For example faster sampling requires brighter probes as is the case of colloidal gold particles, QDs or fluorescent beads. However, use of these typically larger probes is also more artefact prone due to steric effects or probe-induced cross-linking. This was clearly demonstrated already in initial SPT experiments with gold particles [69], and again more recently in live cell studies that compared either colloidal gold and fluorescent dye probes [6], or QDs and fluorescent dye probes [51], and furthermore in a systematic study in supported lipid bilayers that compared the influence of the probe particle as well as the influence of the probe valency [68]. A size comparison of some commonly used SPT probes is shown in figure 1. Most SPT experiments to date have been performed by investigating only one single molecule species at a time but multi-color, multi-species SPT is possible, principally by using QDs, although at the expense of sampling speed [70–72].

#### 2.1.3. Data analysis in SPT.

The most common analysis of SPT trajectories is as discussed based on the theoretical derivation by Einstein that established the theoretical relationship between the MSD, time t, and the diffusion coefficient *D* in one dimension [52, 53]. Typical analysis steps then includes a determination of the centroid position of each single molecule at each time point followed by a step where centroid positions of specific single molecules are linked in time in order to form a series of single molecule trajectories. Starting from these experimentally observed single molecule trajectories it is then possible to extract quantitative information by calculating the MSD as a function of *t*  =  *nt*_lag_, where *t*_lag_ is the characteristics time interval between adjacent camera frames, and *n* is the number of image frames between specific time points. The MSD_expt_(*nt*_lag_) versus *nt*_lag_ data can then be quantified by use of curve fitting to an array of available theoretical diffusion models with a range of incorporated complexities, e.g. the simplest case of Brownian diffusion, confined diffusion, anomalous, diffusion, and combinations thereof (table 1) [15, 39, 73–75]. A very important factor in this analysis is also to use an appropriate number of data points. This is because the variance of the MSD_expt_(*nt*_lag_) increases with larger *n* [73]. Consequently it has been recommended practice to restrict the analysis of MSD_expt_(*nt*_lag_) data to no more than a quarter of the total data points and further to use weighted least-square fits of the data, frequently with weights equal to the inverse variance [45]. But in practice there has been no consensus on which data points, *n*, or alternatively which time points, *n*, *t*_lag_, to use even though this selection is of utmost importance. It has for example been shown by simulations that the accuracy of the extracted diffusion coefficient in the simplest case of free Brownian diffusion in the absence of experimental measurement errors is greatest when only data points 1  ⩽  *n*  ⩽  2 are included in the analysis [39]. Unfortunately, there is no similar comparison for more complicated diffusion processes or in the presence of simulated experimental measurement errors but it follows that the use of more complicated diffusion models also require that a greater number of data points are used in the analysis because these models contain a greater number of free parameters.

**Table 1.** Example of fit models for SPT data.

Fit model	Fit equations $\begin{array}{c} \frac{\text{MS}{{\text{D}}_{\text{expt}}}(n{{t}_{\text{lag}}})}{4n{{t}_{\text{lag}}}}=\frac{\text{MS}{{\text{D}}_{\text{theory}}}(n{{t}_{\text{lag}}})}{4n{{t}_{\text{lag}}}}(1-\frac{2R}{n})+\frac{\Delta _{x,y}^{2}}{n{{t}_{\text{lag}}}} \\ {{D}_{\text{app}}}(n{{t}_{\text{lag}}})=\frac{\text{MS}{{\text{D}}_{\text{theory}}}(n{{t}_{\text{lag}}})}{4n{{t}_{\text{lag}}}} \end{array}$	Calculated parameters	References
1—free diffusion	${{D}_{\text{app}}}\left(n{{t}_{\text{lag}}}\right)={{D}_{\text{MACRO}}}$		[52, 53]
2—anomalous diffusion	${{D}_{\text{app}}}\left(n{{t}_{\text{lag}}}\right)={{D}_{\text{MACRO}}}{{\left(n{{t}_{\text{lag}}}\right)}^{\alpha -1}}$		
3—confined diffusion	${{D}_{\text{app}}}\left(n{{t}_{\text{lag}}}\right)=\frac{{{L}^{2}}}{12n{{t}_{\text{lag}}}}\left(1-\text{Exp}\left[-\frac{n{{t}_{\text{lag}}}}{\tau}\right]\right)$	${{D}_{\mu}}=\frac{{{L}^{2}}}{12\tau}$	[118]
4—confined diffusion within impermeable square corrals	${{D}_{\text{app}}}\left(n{{t}_{\text{lag}}}\right)=\frac{{{L}^{2}}}{12n{{t}_{\text{lag}}}}\left(1-\frac{96}{{{\pi}^{4}}}\sum\nolimits_{k=1\left(\text{odd}\right)}^{\infty}\frac{1}{{{k}^{4}}}\text{Exp}\left[-\frac{{{k}^{2}}{{\pi}^{2}}}{12}\frac{n{{t}_{\text{lag}}}}{\tau}\right]\right)$	${{D}_{\mu}}=\frac{{{L}^{2}}}{12\tau}$	[75]
5—mixed diffusion	${{D}_{\text{app}}}\left(n{{t}_{\text{lag}}}\right)={{D}_{\text{MACRO}}}+\frac{{{L}^{2}}}{12n{{t}_{\text{lag}}}}\left(1-\text{Exp}\left[-\frac{n{{t}_{\text{lag}}}}{\tau}\right]\right)$	${{D}_{\mu}}=\frac{{{L}^{2}}}{12\tau}+{{D}_{\text{MACRO}}}$ ${{S}_{\text{Conf}}}=\frac{{{D}_{\mu}}}{{{D}_{\text{MACRO}}}}$	[5, 15, 74]
6—hop diffusion with permeable periodic squares	$\begin{array}{c} {{D}_{\text{app}}}\left(n{{t}_{\text{lag}}}\right)={{D}_{\text{MACRO}}} \\ +{{\beta}^{2}}\frac{{{L}^{2}}}{12n{{t}_{\text{lag}}}}\left(1-\frac{96}{{{\pi}^{4}}}\sum\nolimits_{k=1(odd)}^{\infty}\frac{1}{{{k}^{4}}}\text{Exp}\left[-\frac{{{k}^{2}}{{\pi}^{2}}}{12\beta}\frac{n{{t}_{\text{lag}}}}{\tau}\right]\right) \end{array}$	${{D}_{\mu}}=\frac{{{\beta}^{2}}{{L}^{2}}}{12\tau}+{{D}_{\text{MACRO}}}$ ${{S}_{\text{Conf}}}=\frac{{{D}_{\mu}}}{{{D}_{\text{MACRO}}}}$	[22]

There are also alternative analysis methods for SPT data. One possibility is to fit to the cumulative probability distribution for square displacements *r*^2^  =  *x*^2^  +  *y*^2^

2 \begin{eqnarray*}{{P}_{\text{theory}}}\left({{r}^{2}},n{{t}_{\text{lag}}}\right)=1-\text{Exp}\left[-\frac{{{r}^{2}}}{r_{0}^{2}}\right]\end{eqnarray*}

where e.g. $r_{0}^{2}$  =  2*dDnt*_lag_ for the case of free diffusion in *d* dimensions [47, 66]. Similar to analysis of FRAP experiments, this fitting approach has also been extended to linear combinations of multiple, discrete, diffusing components i.e. one fast and one slow diffusing component [47, 66]. Recent work have also introduced the use of maximum likelihood estimator [33, 48, 49] and co-variance based estimator [50] approaches for analysis of SPT data. These three latter analysis methods are, in contrast to the MSD analysis, performed separately at each single time point, *nt*_lag_.

There are several possible sources of error in experimental SPT measurements which means that the theoretical derivations for MSD_theory_(*t*) versus *t* dependencies will not match experimentally determined MSD(*nt*_lag_) versus *nt*_lag_ dependencies. First, because diffusion is a stochastic process, the accuracy of the experimentally determined MSD_expt_(*nt*_lag_) curves is directly dependent on the number of observed displacements at each time point *nt*_lag_ [45, 73]. Consequently, accurate analysis can either be performed by ensemble analysis of large set of short single trajectories, as is typically the case for fluorescent dyes and proteins as a result of irreversible photo-bleaching, or by selective analysis of only very long trajectories as can be collected with gold particles or quantum dots.

Other major sources of error in SPT analysis are the limited localization precision of single particles or molecules in each still frame [34, 73] and motion blurring due to finite camera integration times [33, 35, 76]. The measurement error that originates from finite localization precision of single particles or molecules had long been recognized as contributing a positive *y*-offset in MSD versus *t* plots [73] but has only more recently been recognized as a possible cause of anomalous sub-diffusion [34]. The correction term for this has been shown to contribute a positive term to the experimental determined MSD_expt_(*nt*_lag_) versus *nt*_lag_ plots according to

3 \begin{eqnarray*}\text{MS}{{\text{D}}_{\text{expt}}}\left(n{{t}_{\text{lag}}}\right)=2dDn{{t}_{\text{lag}}}+2 \Delta _{r}^{2}\quad \quad \quad \Delta _{r}^{2}= \Delta _{x}^{2}+ \Delta _{y}^{2}+ \Delta _{z}^{2}\end{eqnarray*}

where Δ_*r*_ is the localization error in *r* co-ordinate space where *r*^2^  =  *x*^2^  +  *y*^2^  +  *z*^2^ [34].

The error that stems from motion blurring was initially recognized by Goulian and Simon [76]. This error originates from the fact that a diffusing molecule remains mobile during the finite camera integration time such that all determined centroid positions represent the average particle position. The generic magnitude of this error term is a negative contribution to the MSD_expt_(*nt*_lag_) of

4 \begin{eqnarray*}-4dDR{{t}_{\text{lag}}}\,\quad \quad 0\leqslant R\leqslant 1/4\end{eqnarray*}

where the motion blur coefficient *R* characterizes the illumination profile during the camera integration time, and where e.g. *R*  =  1/6 for the case of full-frame averaging [33, 35]. By combining equations (3) and (4) for the generic case of free diffusion in d dimensions we get

5 \begin{eqnarray*}\text{MS}{{\text{D}}_{\text{expt}}}\left(n\,{{t}_{\text{lag}}}\right)=2dDn\,{{t}_{\text{lag}}}+2 \Delta _{r}^{2}-4dDR{{t}_{\text{lag}}}\end{eqnarray*}

or generically for diffusion in two dimensions where the mode of diffusion is unknown as

6 \begin{eqnarray*}\text{MS}{{\text{D}}_{\text{expt}}}(n{{t}_{\text{lag}}})=\text{MS}{{\text{D}}_{\text{theory}}}\left(n{{t}_{\text{lag}}}\right)\left(1-\frac{2R}{n}\right)+2 \Delta _{r}^{2}\end{eqnarray*}

These corrections have already been implemented for MSD analysis [77], for maximum likelihood estimator [33, 48, 49], and covariance-based estimator [50] approaches, but broad use of these corrected expressions has yet to occur.

Whereas equation (6) is the traditional way of displaying SPT data, it is much more instructive to divide equation (6) for the case of d dimensions by 2*dnt*_lag_ and by defining an apparent diffusion coefficient, *D*_app_(*nt*_lag_)  =  MSD_theory_(*nt*_lag_)/(2*dnt*_lag_) to yield

7 \begin{eqnarray*}\frac{\text{MS}{{\text{D}}_{\text{expt}}}\left(n{{t}_{\text{lag}}}\right)}{2dn{{t}_{\text{lag}}}\left(1-\frac{2R}{n}\right)}={{D}_{\text{app}}}\left(n{{t}_{\text{lag}}}\right)\,+\frac{\Delta _{r}^{2}}{dn{{t}_{\text{lag}}}\left(1-\frac{2R}{n}\right)}\end{eqnarray*}

or for the specific case of two dimensions (*d*  =  2), *R*  =  1/6, and Δ_*x*_  =  Δ_*y*_ and $ \Delta _{r}^{2}$  =  2 $ \Delta _{x,y}^{2}$ as

8 \begin{eqnarray*}\frac{\text{MS}{{\text{D}}_{\text{expt}}}\left(n{{t}_{\text{lag}}}\right)}{4{{t}_{\text{lag}}}\left(n-\frac{1}{3}\right)}={{D}_{\text{app}}}\left(n{{t}_{\text{lag}}}\right)+\frac{\Delta _{x,y}^{2}}{\,{{t}_{\text{lag}}}\left(n-\frac{1}{3}\right)}\end{eqnarray*}

A plot of equation (8) will thus give a direct indication of the time-dependence of the apparent diffusion coefficient *D*_app_(*nt*_lag_). It is then, for example, easy to see that in the simplest case of free Brownian diffusion, no motion blur (*R*  =  0) and no localization error (Δ_*x*,*y*_  =  0), this leads to a constant (time-independent) diffusion coefficient *D*_app_(*nt*_lag_)  =  *D*. Conversely, equation (8) also directly illustrates that a particular challenge in high-speed SPT is that the magnitude of the contribution from the measurement error becomes infinite in the limit of *t*_lag_  →  0. For example, the case of a typical measurement error of Δ_*x*,*y*_  =  25 nm and with *D*  =  1 *µ*m^2^ s^−1^ results in that the magnitude of the error contribution term in equation (8) exceeds the apparent diffusion coefficient term for *t*_lag_  ≲  0.9 ms or for sampling frequencies, 1/*t*_lag_, ≳  1100 Hz.

There are also other potential sources of errors in SPT. This includes in particular membrane ruffling, or possible membrane curvature effects that would result in that the measurement data would no longer match the assumed mathematical models for 2D diffusion [19, 20]. Other error contribution could also come from e.g. internalization of the targeted molecules to the cytosol.

### 2.2. Fluorescence correlation spectroscopy and stimulated emission depletion—fluorescence correlation spectroscopy

#### 2.2.1. Brief description of method.

The raw experimental data in FCS consists of a time-sequence of fluorescence intensity measurements from a fixed focal volume at typical sampling frequencies of about 100 kHz. Nowadays, this is typically accomplished by use of a conventional confocal microscope, equipped with a photon counting detector, e.g. an avalanche photo-diode (APD) or a hybrid gallium arsenide phosphide (GaAsP) detector, by parking the excitation laser to a point of interest and by continuous measurement of the fluorescence intensity from said spot. The focal volume in this instance is defined by using a laser that has been focused to a diffraction limited spot by the microscope objective and by use of a pinhole on the emission side to further restrict the fluorescence signal in the axial direction. This typically results in a diffraction-limited observation spot with a radius of  ≈250 nm, corresponding to an observation volume of  <1 fL. The recorded fluorescence intensity traces are analysed first by calculation of the normalized auto-correlation function *G*(*τ*) (see section 2.3) and second by non-linear curve fitting to an appropriate model that describes the underlying physical process, e.g. diffusion or directed motion of fluorophores through the detection volume, photophysical or photochemical reactions, or on/off binding kinetics [78, 79]. It is very important in FCS to optimize the average number of molecules in the focal volume to relatively dilute conditions between 1 and 1000. This is because the relative intensity fluctuations decrease with increasing numbers of measured fluorophores such that the fluctuations might become indistinguishable from noise. The optimal concentration of the labelled component in FCS thus corresponds to a range from nanomolar (~10^−9^ M) to (sub)micromolar (≲10^−6^ M).

STED (stimulated emission depletion) microscopy is a super-resolution fluorescence approach, which was first introduced in 1994 and allows imaging with in principle unlimited spatial resolution [80]. The application of FCS on a STED microscope (STED-FCS) has several unique advantages [27, 81]. The general approach for STED-FCS is the same as for traditional FCS. Only it also requires a STED laser, red-shifted in wavelength relative to the fluorescence emission of the specific probe, optical elements for focusing the STED laser to a donut pattern with a zero intensity foci in the middle, and exquisite co-alignment in space and time of the excitation and STED lasers [28, 82]. With this approach it has been possible to tune the observation spot from a diffraction-limited value of around 250 nm and down to  ≈15 nm by adjustment of the STED laser power [28, 29]. Because of the significant reduction in the size of the focal volume, the applicable concentration range in STED-FCS is shifted to at least an order of magnitude higher than for conventional FCS.

#### 2.2.2. Development of method.

FCS was first introduced by Magde *et al* [83] as an extension of dynamic light scattering but with a greater magnitude of intensity fluctuations in homogenous media. This technique consists of recording time-sequences of the fluorescence intensity from a fixed sampling volume and by computing the auto-correlation function of the recorded fluorescence signal over the elapsed time of measurement. The correlation function represents the self-similarity of the distribution of molecules in the focal volume over the time and provides information about physicochemical processes undergone by the fluorescent molecules in the relevant time scales. For example, characteristic dark state transitions, binding reactions and diffusion rates are typical processes assessed by the analysis of the autocorrelation function [79]. The technique was first applied to measure the binding of ethidium bromide to DNA [83]. A more detailed theoretical framework followed soon thereafter [84] as did first application to measuring lateral dynamics in re-constituted planar lipid bilayers [85]. Initially limited in applicability due to both technical and biological reasons, FCS gained momentum as a powerful technique in the investigation of a vast range of kinetic processes with the widespread use of confocal microscopy, which allowed single-molecule sensitivity [86]. Subsequently, as fluorescence microscopy underwent a revolution concerning the methods and physical phenomena employed, so did FCS. Two-photon FCS [87], cross-correlation FCS [88], total internal reflection FCS [89, 90], scanning FCS (sFCS) [91, 92], Z-scan FCS [93], and two-focus FCS [94] are among the several versions of FCS that were developed and have helped in the quantification of relevant observables on plasma membranes. Another development has been the introduction of spot-variation (sv)-FCS [95–97], a method that is analogous to FRAP measurements that were obtained as a function of the spot-size [98–100]. Using sv-FCS, it is possible to reveal diffusion patterns based on the relation among diffusion times obtained by FCS recordings at different sizes of the observation area for areas with a size equivalent to a diffraction limited spot and larger [95–97]. The powerful combination of super-resolution stimulated emission depletion (STED) microscopy with FCS has further enabled a direct method for probing membrane dynamics at sub-diffraction volumes [27, 28, 101]. Implementations of scanning STED-FCS, with a lower limit of the focal radius, *ω*_*x*,*y*_, of  ≈80 nm, has also recently been shown [102, 103]. Following the developments of different versions of FCS came also different types of image correlation spectroscopy (ICS, STICS, kICS, RICS) which provide e.g. diffusion information over large areas, but at a compromise of decreased time resolution [104–107]. A promising approach, delivering similar information on molecular diffusion modes (albeit from larger regions of interest), is iMSD, where in the presence of dynamical processes such as diffusion the advanced information content is obtained from probing molecular displacements rather than molecular positions [5, 108, 109]. A more thorough historical account of the development of FCS and its many extensions has been given [110].

#### 2.2.3. Data analysis in FCS.

The raw data in FCS consists of a fluorescence intensity trace that was acquired from an observation volume with a known size. The first step in the analysis is then to calculate the normalized auto-correlation function *G*(*τ*) as a function of the correlation time *τ*

9 \begin{eqnarray*}G\left(\tau \right)=\frac{\langle I(t)I\left(t+\tau \right)\rangle}{{{\langle I(t)\rangle}^{2}}}\end{eqnarray*}

where *I*(*t*) is the intensity at time *t*, *I*(*t*  +  *τ*) is the intensity at *t*  +  *τ*, and $\langle \rangle $ denotes averaging over the measurement time *t*. There now exist any number of theoretical expressions for different physical processes including one- and multi-component expressions for simple Brownian diffusion, anomalous diffusion, photophysical or photochemical reactions, or on/off binding kinetics [78]. An example of this is the expression for a one component anomalous diffusion in two dimensions, which is often used for STED-FCS data [28]

10 \begin{eqnarray*}G\left(\tau \right)=\frac{G(0)}{\left(1+{{\left(\tau /{{\tau}_{D}}\right)}^{\alpha}}\right)}\quad \quad \quad G(0)=\frac{1}{{{V}_{\text{eff}}}\langle C\rangle}\end{eqnarray*}

where *V*_eff_ is the effective focal volume, $\langle C\rangle $ is the mean concentration of the fluorescent species, *τ*_*D*_ is the characteristic correlation time, and *α* is the anomaly coefficient where *α*  =  1 corresponds to simple Brownian diffusion while *α*  <  1 is indicative of e.g. spatially confined diffusion whereas *α*  >  1 is indicative of an active process. The relationship between the characteristic correlation time *τ*_*D*_ and the diffusion coefficient for STED-FCS is given by:

11 \begin{eqnarray*}D=\frac{\omega _{x,y}^{2}}{8\,\ln (2)\,{{\tau}_{D}}}\end{eqnarray*}

Sources of errors in general FCS analysis include optical aberrations that cause deviations in the shape of the observation spot [111]. This is because FCS curves are usually fitted by mathematical models that assume a perfectly Gaussian-shaped observation spot. Consequently, FCS measurements are typically acquired such as to minimize for example refractive index mismatch and other sources of specimen-induced aberrations by selecting measurements points in the membrane that are  ⩽0.25–0.5 *µ*m from the glass coverslip. Extreme care further has to be taken to prevent photo-bleaching of labelled molecules that contribute to the observed fluorescence intensity fluctuations. This is because photo-bleaching in this instance will result in an auto-correlation function with an abnormally fast decay time. Thus it is advisable to always record a series of FCS measurements, at varied laser intensities, to ensure that observed auto-correlation decay times are in fact acquired at settings that are independent of the laser intensities. Like for SPT, measurements errors for FCS may also stem from e.g. internalization of the targeted molecules to the cytosol, membrane ruffling, membrane curvature or membrane folds that would result in measurement data that would no longer match the assumed mathematical models for 2D diffusion [19, 20]. To prevent internalization, a given sample is typically measured for no longer than 30 min. Furthermore, (STED-) FCS measurements are often acquired at the periphery of cells, in regions where the overall distance between apical and basal plasma membranes is much shorter than the full width at half maximum of the point spread function in the *z* direction. Nevertheless, errors in estimated values of the diffusion coefficient, even in the presence of e.g. aberrations, usually amount to less than 50% [112].

Analogous to the case of sv-FCS [95], STED-FCS data is most typically represented by plotting the FCS diffusion law of the resulting diffusion coefficients as a function of the square of the focal radius, *ω*_*x*,*y*_. A pre-requisite of such experiments is then that the dependence of the focal radius on the STED laser power is known for the set-up. This calibration is typically performed from STED-FCS measurements in fluid-phase (DOPC) multilamellar lipid layers containing a trace amount of e.g. Atto647N-DPPE or KK114-DPPE for which it has been shown that *D*  =  5 *µ*m^2^ s^−1^ [28]. This corresponds to an accessible time resolution of STED-FCS for focal radii measurements of 40  ⩽  *ω*_*x*,*y*_  ⩽  250 nm of 60 *µ*s  ⩽  *τ*_*D*_  ⩽  2.3 ms in multi-lamellar lipid layers. The primary error in the STED-FCS data analysis originates from the measurement precision of the lateral radius of the observation spots where typical measurement errors are around 10%, resulting in final errors of the diffusion coefficient of a similar range [28, 29, 82]. The general advantage of this STED-FCS measurement mode is that it mainly relies on comparing relative values (i.e. for different observation sizes), while the absolute values of the diffusion coefficient is of less importance [81].

### 2.3. Bridging data analysis approaches to enable direct comparison of lateral dynamics measurements by SPT and STED-FCS in live cell plasma membranes

#### 2.3.1. Enabling a direct comparison of SPT and STED-FCS data.

The refinement of FCS to enable measurements in smaller observation volumes [113] and the development of SPT for measuring lateral diffusion coefficients of specific single molecules [61, 75, 114] in the late 1980s to early 1990s provided new methods for validating earlier results by FRAP and other techniques as well as for making new discoveries. However, the introduction of high-speed SPT with colloidal gold particles at sampling frequencies up to 50 kHz meant that it was no longer possible to corroborate these findings by any other method or even by another probe. The recent emergence of STED-FCS has meant that studies at comparable spatial and temporal scales of tens of nanometers up to the diffraction limit and at sub-millisecond to tens of milliseconds time scales is possible. This opens up the prospect of directly comparative measurements in the same cell types of the same biomolecules by high-speed SPT and STED-FCS. In particular it would be very advantageous to enable both qualitative and quantitative comparisons of diffusion data in the plasma membrane of live cells in order to resolve current ambiguities related to the confinement strength imposed by the cortical actin cytoskeleton on various membrane constituents. This has not yet been done.

**Figure 2.**
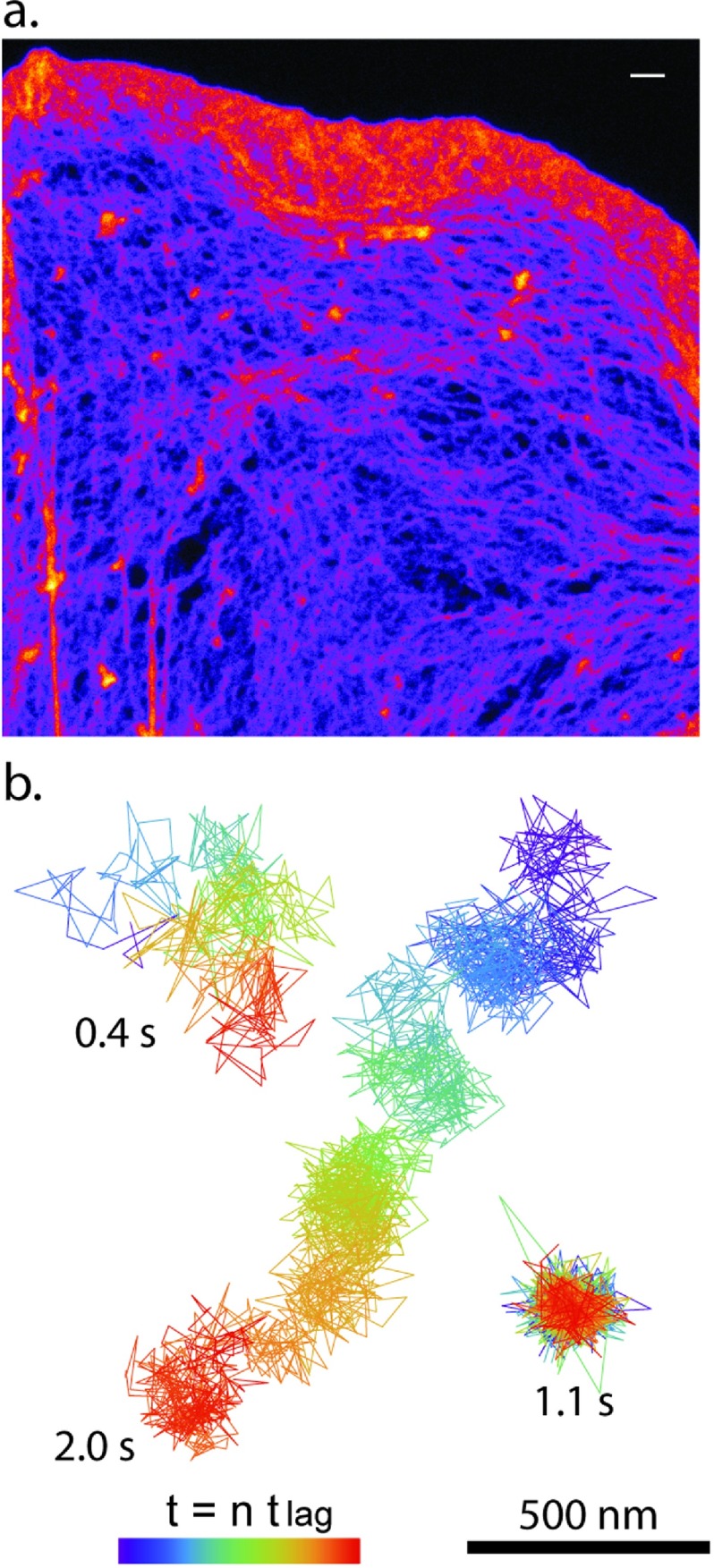
Representative STED image of F-actin structure and SPT trajectories of biotin-cap-DPPE in IA32 mouse embryo fibroblasts. (a) IA32 MEFs were stained for F-actin with Oregon Green 488 phalloidin, mounted in Mowiol, and imaged on a commercial Leica SP8 gated STED microscope. Fluorescence excitation was done with a pulsed white laser (80 MHz) tuned to 514 nm while stimulated emission depletion was done with a CW laser emitting at 592 nm. Time-gated fluorescence emission was collected on a HyD GaAsP detector with a time gate of 1.5  <  *t*  <  6.5 ns with a projected pixel size of 25 nm. The approximate lateral optical resolution of this system at the acquisition settings were 80 nm (scale bar  =  1 *µ*m). (b) Representative trajectories of two mobile and one immobile QD labelled DPPE molecules in IA32 MEFs as observed by high-speed SPT as previously described [23].

To remedy this, we present a direct comparison of SPT [23] and STED-FCS data [24] for the lateral dynamics of a DPPE phospholipid analogue in the plasma membrane of live cells. This data was acquired in the lamella of live Ink4a/Arf (−/−) (IA32) MEFs [115] at RT [24]. A phenotype of the IA32 MEFs is very prominent lamellipodia followed by large, thin lamella (figure 2(a)). A major advantage of SPT is that it is possible to differentiate single molecule behaviour in space and time. This is illustrated by the sample trajectories shown in figure 2(b) which show examples of both mobile and immobile behaviour. Using SPT, it is further also possible to obtain quantitative information from a single molecule provided that the number of data points, *n*, in the trajectory are sufficient for accurate analysis. FCS (or STED-FCS) on the other hand has single molecule detection sensitivity but it is not possible to directly obtain information from a single molecule. Rather using FCS one can obtain a measure of the ensemble average correlation time of all single molecules that diffuse through the observation spot during the observation time which in our measurements was 10 s. Another difference between SPT and FCS measurements is that immobile molecules do typically not contribute to the measurements in FCS because the signal of immobile molecules do not fluctuate but rather are photobleached at the start of the measurements.

To perform this data comparison, we start by noting that whereas the fluorescence intensity data in FCS is collected with point detectors in the time-domain at fixed spatial scales, the fluorescence intensity data in SPT is collected with cameras in the space-domain at fixed time points. Consequently because time measurements can be made with both extreme accuracy and precision, the measurement errors for both methods are dominated by the space-domain. But these errors are manifested in very different ways. The space-domain error in the case of SPT stems from the fact that the centroid position of single molecules can only be determined to within some finite localization error, ±Δ_*r*_, which as a consequence of that the typical SPT analysis first involves the calculation of squared displacements always results in a positive constant offset in MSD_expt_(*nt*_lag_) space (equation (3)). The camera blur error, in contrast, results in a less apparent negative contribution in MSD space (equation (4)) that has the greatest impact on the analysis between adjacent time frames i.e. for points separated by small numbers of image frames *n*. Thus, because the errors in SPT contributes offset terms with an absolute magnitude it is imperative that these errors are directly included in the fitting models as is the case for the expression in equations (6)–(8) as well as in recent work on the development of maximum likelihood estimate approaches for analysing SPT data [48, 50]. The spatial error in STED-FCS, in contrast, introduces an uncertainty in the precision by which the focal radius can be determined. This error subsequently results in a corresponding uncertainty in the calculation of the apparent diffusion coefficient *D*_app_ (usually around 10%) [28, 116], but does not otherwise contribute systematic additive or subtractive terms that must be directly accounted for as is the case for SPT data as discussed above.

#### 2.3.2. Analysis of SPT data.

**Figure 3.**
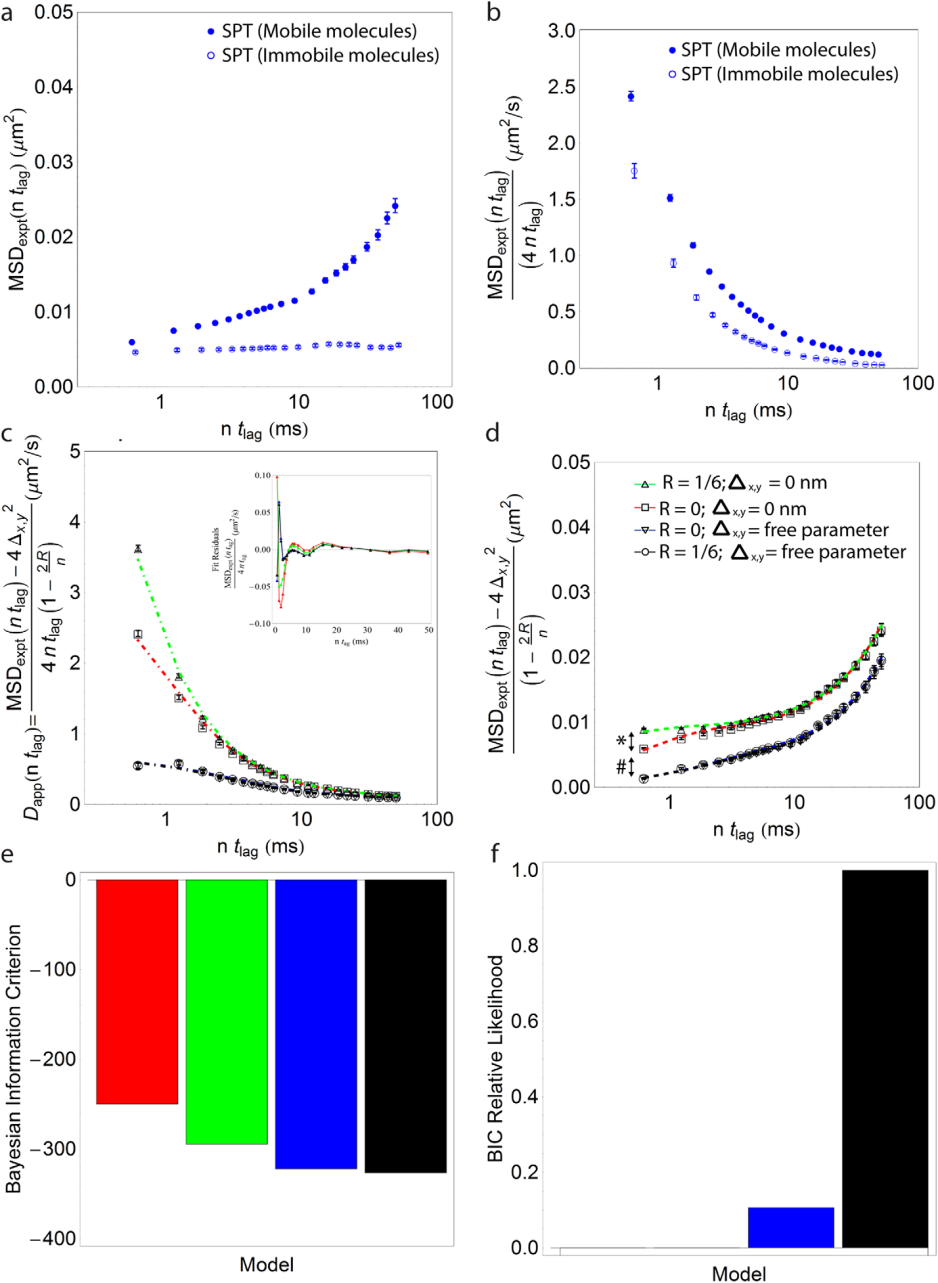
Example of SPT data and fit results to average of SPT trajectories of a phospholipid analogue, biotin-cap-DPPE, labelled with a sAv-QD655s in IA32 MEFs for a time window of 0.6  ⩽  *nt*_lag_  ⩽  50 ms. (a) Separate mean MSD curves for ‘mobile’ (*N*  =  460; closed circles) and ‘immobile’ (*N*  =  33; open circles) molecules. (b) The impact of these errors are more apparent if we convert the raw experimental data from MSD space to apparent pseudo diffusion coefficient, pseudo-*D*_app_ space by plotting MSD_expt_(*nt*_lag_)/(4*nt*_lag_) versus *nt*_lag_.This results in that the raw experimental data has the appearance of a time-dependent apparent diffusion coefficient, in these cases ranging for the case of mobile molecules from an upper range of  ≈2.5 *µ*m^2^ s^−1^ and down to  ≈0.1 *µ*m^2^ s^−1^ and for immobile molecules from an upper range of  ≈1.8 *µ*m^2^ s^−1^ and down to  ≈0 *µ*m^2^ s^−1^. To distinguish the contributions to the data that stems from the errors, the data in the format shown in (b) for the mobile trajectories was fit to a mixed diffusion model (model 5 in table 1) for a various time windows with and without a correction for motion blur (*R*  =  0 or *R*  =  1/6) and either in the absence of localization noise (Δ_*x*,*y*_  =  0 nm), or where the localization noise was a free parameter of the fit. The resulting weighted least-square fits, where the data point was weighted according of the inverse variance, are shown in (c) for the cases where the data is corrected for motion blur but not localization error (*R*  =  1/6 and Δ_*x*,*y*_  =  0; green dashed line, open triangle), data is neither corrected for motion blur nor localization error (raw data case; *R*  =  0 and Δ_*x*,*y*_  =  0; red dashed line, open square), data is corrected for localization noise but not motion blur (*R*  =  0 and Δ_*x*,*y*_ as a free parameter; blue dashed line, open circle), and data is corrected for both motion blur and localization noise (*R*  =  1/6 and Δ_*x*,*y*_ as a free parameter; black dashed line, inverted open triangle). The numerical results of the weighted fits for four different time windows, 0.6  ⩽  *nt*_lag_  ⩽  *t*_end_ where *t*_end_  =  5, 10, 25, and 50 ms, are shown in table 2 while the results for the unweighted fits are shown in table 3. Also shown in (d) are the results of the fits for the same cases as in (c) but in error corrected MSD space. The effect on the uncorrected MSD curve (open square; red dashed line) that stems from the motion blur correction (*) and the correction that stems from the localization error correction (#) are furthermore explicitly shown in (d). A critical aspect when comparing different models is to have an efficient means for evaluating the performance of the different models; one such possibility is to determine the Bayesian Information Criteria (BIC) for the performance of each model (e) and subsequently to use the BIC values for each model to calculate the relative likelihood of the suitability of each model (f).

To explore the error contributions in our SPT measurements, we have plotted the mean, raw, MSD curves for the observed motion of a phospholipid analogue, biotin-cap-DPPE, labelled with a streptavidin (sAv) conjugated quantum dot with peak emission at 655 nm (QD655) (figure 3(a)) that was acquired with a sampling frequency of  ≈1.8 kHz at RT as described previously [23]. In this plot we have further differentiated average, raw, MSD data for mobile molecules (defined by MSD_Max_(*nt*_lag_)  −  MSD_Min_(*nt*_lag_)  >  4 (25 nm)^2^; *N*  =  460 trajectories; equation (3)) and immobile molecules (defined by MSD_Max_(*nt*_lag_)  −  MSD_Min_(*nt*_lag_)  ⩽  4 (25 nm)^2^; *N*  =  33 trajectories; equation (3)). By differentiating mobile and immobile molecules, we are further able to obtain a better comparison to the STED-FCS data where immobile particles as discussed do not contribute.

**Table 2.** Weighted fit analysis results of SPT data in IA32 MEFs with fit weights of (*n*/*δ*_MSD_)^2^.

	R	*D*_MACRO_ (*µ*m^2^ s^−1^)	*L*^2^/(12*τ*) (*µ*m^2^ s^−1^)	*L* (nm)	Δ_*x*,*y*_ (nm)	*τ* (ms)	${{D}_{\mu}}$ (*µ*m^2^ s^−1^)	BIC relative likelihood
0.6 ⩽ *nt*_lag_ ⩽ 50 ms	0	0.083^a^ ± 0.001^b^ (*p* < 0.001^c^)	3.7 ± 0.1 (*p* < 0.001)	159 ± 0.7 (*p* < 0.001)	—	0.6 ± 0.0	3.7 ± 0.1	0
	1/6	0.080 ± 0.001 (*p* < 0.001)	16 ± 5 (*p* < 0.001)	163 ± 0.4 (*p* < 0.001)	—	0.1 ± 0.0	16 ± 5	0
	0	0.079 ± 0.001 (*p* < 0.001)	0.61 ± 0.06 (*p* < 0.001)	113 ± 2 (*p* < 0.001)	34.0 ± 0.6 (*p* < 0.001)	1.7 ± 0.2	0.69 ± 0.06	0.11
	1/6	0.078 ± 0.001 (*p* < 0.001)	0.62 ± 0.06 (*p* < 0.001)	110 ± 2 (*p* < 0.001)	35.7 ± 0.5 (*p* < 0.001)	1.6 ± 0.2	0.70 ± 0.06	1

0.6 ⩽ *nt*_lag_ ⩽ 25 ms	0	0.095 ± 0.002 (*p* < 0.001)	3.9 ± 0.1 (*p* < 0.001)	155 ± 0.8 (*p* < 0.001)	—	0.5 ± 0.0	4.0 ± 0.1	0
	1/6	0.088 ± 0.001 (*p* < 0.001)	20 ± 14^d^ (*p* = 0.17)	161 ± 0.5 (*p* < 0.001)	—	0.1 ± 0.1	20 ± 14	0.48
	0	0.090 ± 0.002 (*p* < 0.001)	0.92 ± 0.1 (*p* < 0.001)	114 ± 3 (*p* < 0.001)	31.8 ± 1 (*p* < 0.001)	1.2 ± 0.2	1.0 ± 0.1	0.65
	1/6	0.088 ± 0.001 (*p* < 0.001)	0.92 ± 0.1 (*p* < 0.001)	107 ± 2 (*p* < 0.001)	34.5 ± 0.7 (*p* < 0.001)	1.0 ± 0.2	1.0 ± 0.1	1

0.6 ⩽ *nt*_lag_ ⩽ 10 ms	0	0.11 ± 0.007 (*p* < 0.001)	4.1 ± 0.2 (*p* < 0.001)	152 ± 2 (*p* < 0.001)	—	0.5 ± 0.0	4.2 ± 0.2	0.001
	1/6	0.089 ± 0.004 (*p* < 0.001)	20 ± 20^d^ (*p* = 0.32)	161 ± 1 (*p* < 0.001)	—	0.1 ± 0.1	20 ± 20	0.28
	0	0.081 ± 0.01 (*p* < 0.001)	0.82 ± 0.2 (*p* < 0.001)	115 ± 3 (*p* < 0.001)	32.5 ± 1 (*p* < 0.001)	1.3 ± 0.2	0.91 ± 0.2	0.82
	1/6	0.080 ± 0.009 (*p* < 0.001)	0.80 ± 0.1 (*p* < 0.001)	109 ± 3 (*p* < 0.001)	35.0 ± 0.7 (*p* < 0.001)	1.2 ± 0.2	0.88 ± 0.1	1

0.6 ⩽ *nt*_lag_ ⩽ 5 ms	0	0.17 ± 0.004 (*p* < 0.001)	4.6 ± 0.1 (*p* < 0.001)	145 ± 1 (*p* < 0.001)	—	0.4 ± 0.0	4.7 ± 0.1	1
	1/6	0.096 ± 0.01 (*p* = 0.001)	20 ± 28^d^ (*p* = 0.51)	161 ± 2 (*p* < 0.001)	—	0.1 ± 0.2	20 ± 28	0
	0	0.16 ± 0.007 (*p* < 0.001)	3.5 ± 1 (*p* = 0.03)	134 ± 11 (*p* < 0.001)	16.1 ± 8 (*p* = 0.13)	0.4 ± 0.1	3.6 ± 1	0.16
	1/6	0.14 ± 0.01 (*p* < 0.001)	3.9 ± 6.4^d^ (*p* = 0.57)	118 ± 35 (*p* = 0.03)	27.6 ± 11 (*p* = 0.07)	0.3 ± 0.5	4.0 ± 6	0.087

a Fit parameter estimate.

b Standard error of the estimate.

c The *p*-values are the two-sided *p*-values for each parameter calculated from the *t*-statistics, which in turn was calculated as the fit parameter estimates divided by the standard errors. This *p*-value can then be used to assess whether the parameter estimate is statistically significantly different from 0.

d It is imperative that a suitable theoretical model for experimental data not only provides a good overall fit to the data but also that each parameter within the model converges to a well defined value at a selected *p*-value (e.g. *p* < 0.05). While the overall fit of a model can be evaluated by the BIC, the convergence of each parameter can be observed by separate inspection of the *p*-value for each parameter.

**Table 3.** Unweighted fit analysis results of SPT data in IA32 MEFs.

	R	*D*_MACRO_ (*µ*m^2^ s^−1^)	*L*^2^/(12 *τ*) (*µ*m^2^ s^−1^)	*L* (nm)	Δ_*x*,*y*_ (nm)	*τ* (ms)	${\lim\nolimits_{n{{t}_{\text{lag}}}\to 0}}{{D}_{\text{app}}}\left(n{{t}_{\text{lag}}}\right)$ (*µ*m^2^ s^−1^)	BIC relative likelihood
0.6 ⩽ *nt*_lag_ ⩽ 50 ms	0	0.092^a^ ± 0.002^b^ (*p* < 0.001^c^)	4.2 ± 0.1 (*p* < 0.001)	153 ± 0.6 (*p* < 0.001)	—	0.5 ± 0.0	4.3 ± 0.06	0
	1/6	0.082 ± 0.001 (*p* < 0.001)	20 ± 5 (*p* < 0.001)	162 ± 0.3 (*p* < 0.001)	—	0.1 ± 0.0	20 ± 5	0
	0	0.084 ± 0.001 (*p* < 0.001)	1.1 ± 0.07 (*p* < 0.001)	119 ± 1 (*p* < 0.001)	30.5 ± 0.5 (*p* < 0.001)	1.1 ± 0.1	1.2 ± 0.07	0.006
	1/6	0.083 ± 0.001 (*p* < 0.001)	1.1 ± 0.08 (*p* < 0.001)	111 ± 1 (*p* < 0.001)	33.7 ± 0.3 (*p* < 0.001)	0.9 ± 0.1	1.2 ± 0.08	1

0.6 ⩽ *nt*_lag_ ⩽ 25 ms	0	0.11 ± 0.003 (*p* < 0.001)	4.3 ± 0.1 (*p* < 0.001)	151 ± 0.7 (*p* < 0.001)	—	0.5 ± 0.0	4.4 ± 0.1	0
	1/6	0.086 ± 0.002 (*p* < 0.001)	20 ± 6 (*p* = 0.003)	162 ± 0.4 (*p* < 0.001)	—	0.1 ± 0.0	20 ± 6	0
	0	0.095 ± 0.002 (*p* < 0.001)	1.4 ± 0.1 (*p* < 0.001)	119 ± 2 (*p* < 0.001)	29.0 ± 0.8 (*p* < 0.001)	0.9 ± 0.1	1.5 ± 0.1	0.06
	1/6	0.093 ± 0.002 (*p* < 0.001)	1.4 ± 0.1 (*p* < 0.001)	110 ± 1 (*p* < 0.001)	32.8 ± 0.5 (*p* < 0.001)	0.7 ± 0.1	1.5 ± 0.1	1

0.6 ⩽ *nt*_lag_ ⩽ 10 ms	0	0.13 ± 0.007 (*p* < 0.001)	4.4 ± 0.1 (*p* < 0.001)	148 ± 1 (*p* < 0.001)	—	0.4 ± 0.0	4.5 ± 0.1	0.022
	1/6	0.084 ± 0.007 (*p* < 0.001)	20 ± 11^d^ (*p* = 0.099)	162 ± 1 (*p* < 0.001)	—	0.1 ± 0.1	20 ± 11	0.003
	0	0.11 ± 0.008 (*p* < 0.001)	1.8 ± 0.4 (*p* < 0.001)	121 ± 4 (*p* < 0.001)	26.6 ± 2 (*p* < 0.001)	0.7 ± 0.1	1.9 ± 0.4	0.27
	1/6	0.11 ± 0.007 z(*p* < 0.001)	2.0 ± 0.5 (*p* = 0.003)	112 ± 4 (*p* < 0.001)	31.3 ± 1 (*p* < 0.001)	0.5 ± 0.1	2.1 ± 0.5	1

0.6 ⩽ *nt*_lag_ ⩽ 5 ms	0	0.17 ± 0.003 (*p* < 0.001)	4.6 ± 0.0 (*p* < 0.001)	145 ± 0 (*p* < 0.001)	—	0.4 ± 0.0	4.7 ± 0.03	1
	1/6	0.077 ± 0.02 (*p* = 0.008)	58 ± 8 × 10^5^^d^ (*p* = 1)	162 ± 2 (*p* < 0.001)	—	0.038 ± 500	58 ± 8x10^5^	0
	0	0.16 ± 0.007 (*p* < 0.001)	3.8 ± 0.9 (*p* = 0.01)	138 ± 8 (*p* < 0.001)	13.2 ± 8 (*p* = 0.16)	0.4 ± 0.1	4.0 ± 0.9	0.075
	1/6	0.13 ± 0.009 (*p* < 0.001)	4.1 ± 5.6^d^ (*p* = 0.50)	121 ± 29 (*p* = 0.01)	27.1 ± 10 (*p* = 0.05)	0.3 ± 0.4	4.3 ± 6	0.008

a Fit parameter estimate.

b Standard error of the estimate.

c The *p*-values are the two-sided *p*-values for each parameter calculated from the *t*-statistics, which in turn was calculated as the fit parameter estimates divided by the standard errors. This *p*-value can then be used to assess whether the parameter estimate is statistically significantly different from 0.

d It is imperative that a suitable theoretical model for experimental data not only provides a good overall fit to the data but also that each parameter within the model converges to a well defined value at a selected *p*-value (e.g. *p* < 0.05). While the overall fit of a model can be evaluated by the BIC, the convergence of each parameter can be observed by separate inspection of the *p*-value for each parameter.

**Figure 4.**
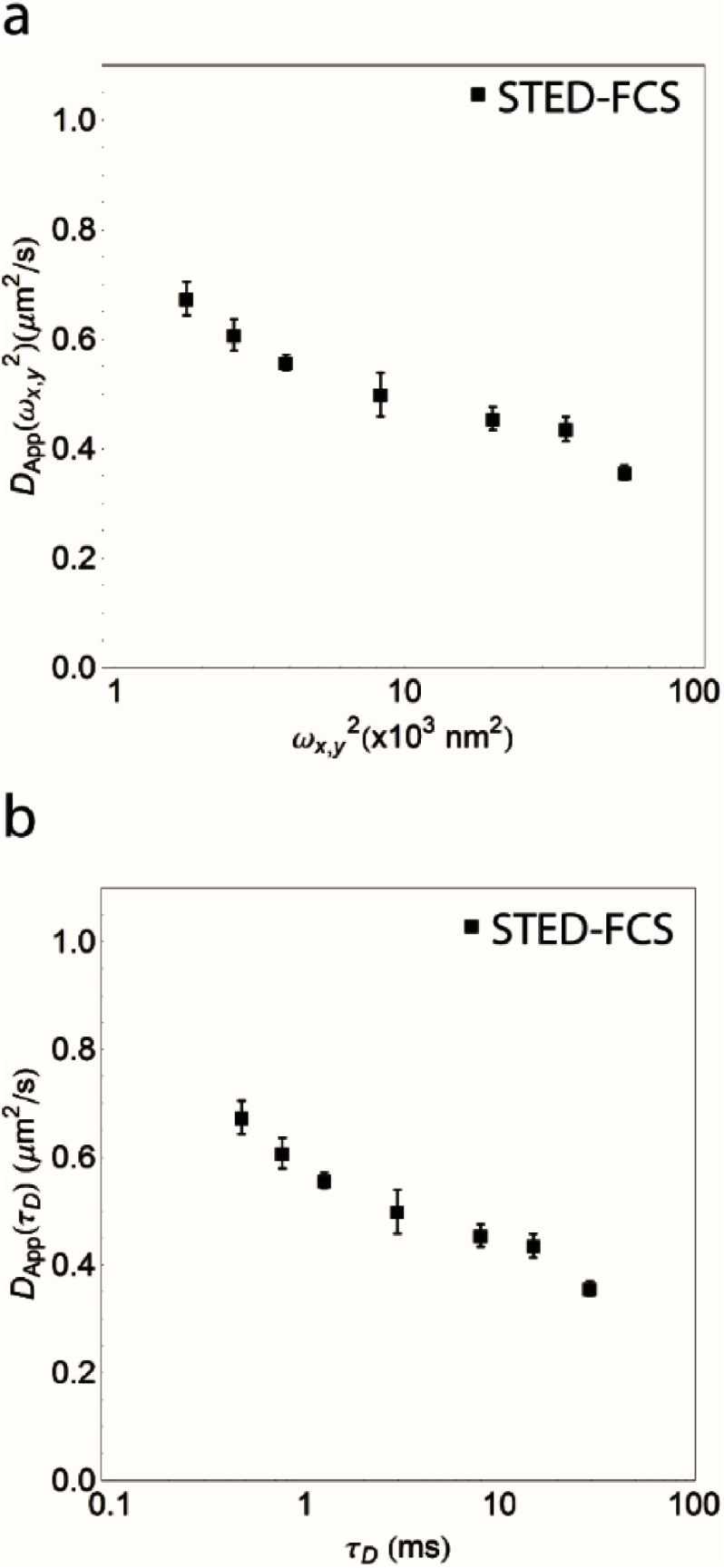
Visualization of FCS diffusion law for DPPE analogues in IA32 MEFs. The traditional display of the FCS diffusion law is to plot the apparent diffusion coefficient *D*_app_ as a function of the square of the focal radius. In the case of free diffusion, this plot would yield an apparent diffusion coefficient *D*_app_ that is independent of the focal radius whereas a hindered diffusion mode such as hop-diffusion would yield a declining curve as shown where *D*_app_ is greater at smaller focal radii than at larger focal radii. In contrast, ‘trapping’ as found for e.g. GSLs in Ptk2 cells [28] would result in a curve *D*_app_ is lower at small focal radii and greater at larger focal radii. An alternative visualization of the same data is shown in (b) where we have instead plotted the dependence of *D*_app_ as a function of the correlation time *τ*_*D*_. The expected behaviour of the data in this format is the same as in (a) that the case of free diffusion would result in a constant *D*_app_ independent of the correlation time whereas the decaying trend as shown is indicative of hindered diffusion. One advantage of the display format in (b) is that this directly shows the range of the observed correlation times, *τ*_*D*_, in the measured system.

The presence of error in the MSD curves in figure 3(a) is directly apparent from the curves, both for the mobile and immobile case, which as expected deviate positively in the direction of the *y*-axis where the theoretical offset in this case of diffusion in 2D is 4Δ_*x*,*y*_ (equation (6)). The impact of these errors becomes more apparent upon converting the data from the traditional MSD versus time format (‘MSD space’) to the alternate MSD/(4*nt*_lag_) versus time format (‘MSD/*t* versus *t* space’) (equations (7) and (8)) which can effectively also be thought of as a pseudo apparent diffusion coefficient space (‘pseudo-*D*_app_ space’) which provides an accurate view of the real *D*_app_ (*nt*_lag_) dependency at long times but that is dominated by measurement errors at very short times. In particular, we see that the plots in MSD/*t* versus *t* space diverges in the limit of *nt*_lag_  →  0 as a consequence of that the error contribution, Δ_*x*,*y*_/(*nt*_lag_), diverges. This is the case for both mobile and immobile trajectories. Consequently, the raw experimental data has the appearance of a time-dependent pseudo-*D*_app_(*nt*_lag_) relation, in the case for mobile trajectories ranging from 2.5 *µ*m^2^ s^−1^ down to  ≈0.1 *µ*m^2^ s^−1^, and in the case of immobile trajectories from an upper range of 1.8 *µ*m^2^ s^−1^ and down to  ≈0 *µ*m^2^ s^−1^. The divergence of the SPT raw data in MSD/*t* versus *t* space, even for molecules that have been identified as immobile, hints strongly that extreme care has to be taken in particular for SPT analysis in the limit where the sampling frequency is very large as directly illustrated in figure 4(b) and by equation (8).

In order to distinguish the real diffusive contribution from the measurement errors, we subsequently fit the raw data for the mobile trajectories in MSD/*t* versus *t* space by least-square fitting in Wolfram Mathematica 9 as shown in figure 4(b) to mixed diffusion model 5 in table 1 [74, 117]. This model combines a time-independent diffusion component *D*_MACRO_ and a time-dependent component (*L*^2^/(12*τ*))(1  −  Exp[−*t*/*τ*]). Fits were performed with all possible combinations of correction for motion blur (*R*  =  0 or *R*  =  1/6) and correction for localization error (Δ_*x*,*y*_  =  0 or Δ_*x*,*y*_ as a free parameter in the fit). We performed both weighted fits, where data points where weighted according to the normalized inverse variance, and unweighted fits for four characteristic time windows 0.6  ⩽  *nt*_lag_  ⩽  *t*_end_ where *t*_end_  =  5, 10, 25 or 50 ms. In these fits, it is worth noting that the weighing of the data points by the inverse variance in MSD/*t* versus *t* space has the consequence that more sampling weight is placed on points that are separated by a larger number of image frames, *n*, as opposed to points from shorter frame separations, *n*, that are more susceptible to both the influence of the camera blur and the localization error as shown in equation (8). The results of the corrected weighted fits for time points of 0.6  ⩽  *nt*_lag_  ⩽  50 ms in error-corrected apparent diffusion space by solving equation (8) for *D*_app_(*nt*_lag_) are shown in figure 3(c) and the numerical fit results for all fits are shown in table 2 (weighted fit), and 3 (unweighted fit). From figure 3(c) and the results in tables 2 and 3. The results of the fits for the same cases but in error-corrected MSD space by solving equation (7) for MSD_theor*y*_(*nt*_lag_) are also shown in figure 3(d).

#### 2.3.3. Statistical comparison of SPT models.

An absolute requirement for fitting SPT data should also be a statistical mean for comparing the applicability of different models. We have previously used F-statistics to classify SPT trajectories but this approach requires the use of nested models [23]. In the work presented here we have instead used the Aikaike information criterion (AIC) [118] respectively the Bayesian information criterion (BIC) [119] to evaluate the suitability of each model. Using this approach, the lower the magnitude of the AIC or BIC the better is the fit of the model to the data but where the difference between the two methods is that the BIC generally penalizes free parameters more strongly than the AIC. These approaches do not require that the tested models are nested but they do require that the numerical values of the dependent variable are identical for all estimates being compared. Thus, we can only use this approach to determine the best model for a specific time window and not to determine e.g. the best time window to use for the analysis. Using the AIC and the BIC it is also possible to calculate the relative likelihood of each model from

12 \begin{eqnarray*}\text{Relative}\,\text{Likelihood}\,=\,\text{Exp}\left[\frac{(A\text{/}B)\text{I}{{\text{C}}_{\text{Min}}}(\text{model})\,-\,(A/B)\text{IC(model})}{\text{2}}\right]\end{eqnarray*}

#### 2.3.4. Results of statistical comparison of SPT models.

The results for the AIC and BIC for the presented data were quantitatively similar; consequently we only show the results for the BIC in figure 3(e) and the relative likelihood of the four versions of the fit model in figure 3(f). This indicates that the most likely version of the mixed diffusion model for the mobile trajectory data, for a time window of 0.6  ⩽  *nt*_lag_  ⩽  50 ms, is the version that corrects for camera blur (*R*  =  1/6) and where the localization error was determined directly from the fit. The best estimate of the definitive physical parameters for this case were Δ_*x*,*y*_  =  35.7  ±  0.5 nm, *D*_MACRO_  =  0.078  ±  0.001 *µ*m^2^ s^−1^, *D*_*μ*_  =  0.70  ±  0.06 *µ*m^2^ s^−1^, and *L*  =  110  ±  2 nm, resulting in a confinement time within confinement regions of *τ*_confinement_  =  *L*^2^/(4*D*_MACRO_)  =  39  ±  1 ms and a confinement strength, *S*_Conf_ of  ≈9. The same diffusion model was also determined to be the most likely, as determined from the BIC relative likelihood (BIC RL), for time windows of 0.6 ms  ⩽  *nt*_lag_  ⩽  *t*_end_ ms with *t*_end_  =  10 or 25 ms. The fit parameters were very similar to those listed above where in particular the short term diffusion coefficient *D*_*μ*_ was in the range of  ≈0.7–1.0 *µ*m^2^ s^−1^ and the confinement strength *S*_Conf_ was  ≈9–11 (table 2). In addition, the fit parameters from the model that did not correct for camera blur (i.e. *R*  =  0) but which included the localization error, Δ_*x*,*y*_, as a free parameter of the fit also resulted in very similar fit estimates except that the localization error values were marginally smaller (table 2). This model also increased in BIC RL as the time window decreased such that it was 0.11 for *t*_end_  =  50 ms, 0.65 for *t*_end_  =  25 ms, and 0.82 for *t*_end_  =  10 ms. We suggest that the observed convergence of the parameter estimates from the fits at time windows of 0.6 ms  ⩽  *nt*_lag_  ⩽  *t*_end_ ms with *t*_end_  =  10, 25, and 50 ms, strongly validates both our analysis approach as well as the extracted fit parameter with a size of the confinement region, *L*, of  ≈110–115 nm, a short term diffusion coefficient *D*_*μ*_ of  ≈0.7–1.0 *µ*m^2^ s^−1^, and a confinement strength *S*_Conf_ of  ≈9–11. By performing a comparison of all the remaining diffusion models in table 1, we further validated that the mixed diffusion model 5 in table 1 was indeed the most likely model for the trajectory data from mobile molecules. The BIC relative likelihood comparison for this analysis confirmed that mixed diffusion model 5 was the most likely model for time windows of 0.6  ⩽  *nt*_lag_  ⩽  *t*_end_ where *t*_end_ was 10, 25, or 50 ms (data not shown).

The most likely model for the shortest time window of 0.6 ms  ⩽  *nt*_lag_  ⩽  5 ms, (data points 1  ⩽  *n*  ⩽  8) was in contrast the model that did neither correct for camera blur (*R*  =  0) nor localization error (Δ_*x*,*y*_  =  0 nm). The results of the corresponding fit was a slightly larger confinement region size of *L*  =  145  ±  1 nm, a much larger *D*_*μ*_  =  4.7  ±  0.1 *µ*m^2^ s^−1^ and a two fold greater *D*_MACRO_  =  0.17  ±  0.004 *µ*m^2^ s^−1^. This model is thus indicative of very fast diffusion within the confinement region and of a three fold larger confinement strength of  ≈27 (as compared to the analysis for longer time windows). However, application of the same model to the data for the immobile trajectories, for the same shortest time window of 0.6 ms  ⩽  *nt*_lag_  ⩽  5 ms, resulted in fit parameters of *L*  =  120  ±  0.2 nm, *D*_*μ*_  =  5.4  ±  0.1 *µ*m^2^ s^−1^ and *D*_MACRO_  =  0.016  ±  0.001 *µ*m^2^ s^−1^. This thus also correspond to a model, where even molecules that had been pre-defined as immobile are diffusing very fast within a confinement region but with an even greater confinement strength of  ≈340. In contrast, a fit of the same immobile trajectory data for the longer time window of 0.6 ms  ⩽  *nt*_lag_  ⩽  50 ms resulted in that the most likely model was one that corrected for camera blur (*R*  =  1/6) and with the localization error as a free parameter of the fit. This fit resulted in Δ_*x*,*y*_  =  33.8  ±  0.6 nm, with a smaller confinement region of *L*  =  51  ±  5 nm, a much lower *D*_*μ*_  =  0.058  ±  0.027 *µ*m^2^ s^−1^, and a *D*_MACRO_  =  0.000  ±  0.001 *µ*m^2^ s^−1^ that is characteristic of an immobile molecule. This thus corresponds to infinite confinement strength. The difference in these results is thus that the fits for shorter time windows preferentially interpret the localization error as a fast diffusive component, *D*_*μ*_, within a confining region whereas fits to longer time windows results in an interpretation of that the diffusive component is either close to immobile (for the case of trajectories that were pre-determined as immobile based on the observed range of the mean squared displacement) or are in the case of the mobile trajectories in this example in the range of *D*_*μ*_  ≈  0.7–1.0 *µ*m^2^ s^−1^ (depending on the exact time window of the analysis). The above results (from the analysis of very short time windows) directly illustrate a possible problem that can originate in such fits. This is a consequence of that data points that are separated by only a few image frames, *n*, are much more susceptible to both the influence of the camera blur and the localization error, as shown by equations (6)–(8). This can easily lead to an over-estimation of the short-term, intra-compartment, diffusion coefficient, *D*_*μ*_, at the expense of an under-estimation of in particular the localization error Δ_*x*,*y*_. This is especially true for data that is acquired at very fast sampling frequencies, 1/(*nt*_lag_), and results from the divergence of the error contribution Δ_*x*,*y*_/(*nt*_lag_) in the limit of *nt*_lag_  →  0.

We also performed an unweighted fit of the same data and time windows (table 3). The results for the BIC relative likelihood for the respective models, for the same time windows, were identical for the cases above for the weighted fit. The fit values, for time windows of 0.6  ⩽  *nt*_lag_  ⩽  *t*_end_ ms with *t*_end_  =  10, 25 or 50 ms resulted in similar compartment sizes of  ≈110 nm, and *D*_MACRO_ of  ≈0.08–0.11 *µ*m^2^ s^−1^. The localization errors were, however, consistently lower by 2–3 nm and the values for *D*_*μ*_ steadily increased for decreasing time windows from  ≈1.2 *µ*m^2^ s^−1^ with *t*_end_  =  50 ms, to  ≈1.5 *µ*m^2^ s^−1^ with *t*_end_  =  25 ms, and  ≈2.1 *µ*m^2^ s^−1^ with *t*_end_  =  10 ms (table 3). The confinement strength consequently increased successively from  ≈14 at *t*_end_  =  50 ms to  ≈20 at *t*_end_  =  10 ms. The most likely model for the shortest time window of 0.6  ⩽  *nt*_lag_  ⩽  5 ms was again the model that did neither correct for camera blur (*R*  =  0) nor localization error (Δ_*x*,*y*_  =  0 nm), and the results for the fit were effectively equivalent to the comparative weighted fit (table 2). This again pinpoints what seems to be a general problem of the SPT analysis in that placing too much emphasis on data points that are acquired at small frame intervals, *n*, can easily lead to an over-estimation of the short-range diffusion coefficient *D*_*μ*_.

#### 2.3.5. Quantitative comparison of STED-FCS data and SPT data.

To pursue this further, we have re-evaluated previously published STED-FCS data [24]. This STED-FCS data that was acquired in the same cell type (IA32 MEFs), for the same phospholipid (DPPE), but where the phospholipid analogue in this case was a head-group labelled Atto647N-DPPE (as opposed to the head-group biotinylated biotin-cap-DPPE that was used for the SPT data in figure 3). The STED-FCS analysis showed that *D*_*μ*_  ≈  0.8  ±  0.03 *µ*m^2^ s^−1^, *L*  ≈  150  ±  12 nm and *D*_MACRO_  ≈  0.4 *µ*m^2^ s^−1^, resulting in a confinement time of *τ*_confinement_  =  *L*^2^/(4*D*_MACRO_)  =  14  ±  2 ms and a confinement strength, *S*_Conf_  ≈  2 [24]. The STED-FCS data (figure 4(a)) thus agrees very closely with the error-corrected SPT data (figure 3(c)) with respect to the short-range, intra-compartment, diffusion coefficient *D*_*μ*_ of  ≈0.7–1.0 *µ*m^2^ s^−1^ from SPT from analysis of time windows ranging from 0.6  ⩽  *nt*_lag_  ⩽  *t*_end_ ms with *t*_end_  =  10, 25 or 50 ms versus  ≈0.8 *µ*m^2^ s^−1^ for STED-FCS. The compartment sizes (≈110 nm for SPT versus  ≈150 nm for STED-FCS) are also in close agreement, but the long-range, inter-compartment, diffusion coefficient, *D*_MACRO_, was five fold smaller for the SPT data (≈0.078 *µ*m^2^ s^−1^) than for the STED-FCS data (0.4 *µ*m^2^ s^−1^). Similarly the confinement strength for the SPT data was almost five fold stronger than for the STED-FCS data.

#### 2.3.6. Enabling a direct qualitative comparison of SPT and STED-FCS data.

It would also be very advantageous to be able to directly compare the SPT and STED-FCS data qualitatively. To do this we can convert the traditional STED-FCS diffusion law data (i.e. plotting of the dependence of the apparent diffusion coefficient, *D*_app_, on the square of the focal radius, $\omega _{x,y}^{2}$ (figure 4(a))) to a format that can be directly compared to the presented SPT data in figure 3(d). One such possibility would be to instead plot the dependence of the apparent diffusion coefficient, *D*_app_, as a function of time as for the case of the SPT data in figure 3(d) but where the time in this case is the characteristic correlation time *τ*_*D*_ (figure 4(b)). The theoretical expected dependence in this data format is the same as in figure 4(a) such that the case of free diffusion would result in a constant *D*_app_ independent of the correlation time whereas the decaying trend as shown is indicative of a hindered diffusion mode such as a hop-diffusion like mechanism. One advantage of the display format in figure 4(b) is also that this directly shows the range of the observed characteristic correlation times, *τ*_*D*_, in the measured system. In the example shown here the time range was  ≈0.5  ⩽  *τ*_*D*_  ⩽≈30 ms for the shown cellular measurements for the pre-determined range of observation radii of  ≈40  ⩽  *ω*_*x*,*y*_  ⩽  240 nm. This time range of cellular measurements is thus directly comparable to the SPT data in figure 3(d). In contrast, the corresponding accessible time range from calibration measurements of a freely diffusing lipid analogue in a SLB was  ≈60 *µ*s  ⩽  *τ*_*D*_  ⩽  2.3 ms (data not shown).

**Figure 5.**
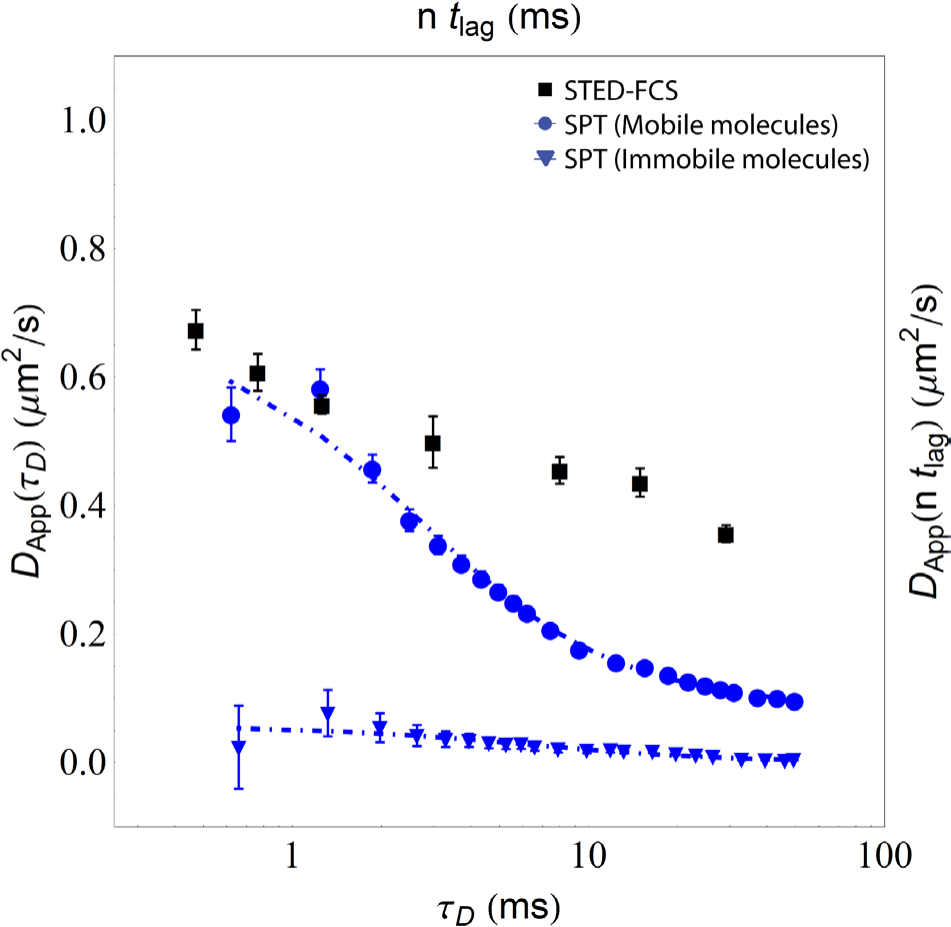
Superposition of STED-FCS data and SPT data, which has been corrected for camera blur and localization error artefacts, for diffusion of DPPE analogues in IA32 MEFs. The super-imposed data STED-FCS (black squares) and mobile SPT data (blue filled circles) agrees closely with an apparent diffusion coefficient, *D*_app_  ≈  0.7 *µ*m^2^ s^−1^, at short times of *t*  ⩽  ≈2 ms, corresponding to diffusion within confining domains whereas the SPT data at longer times is much slower. In contrast, the apparent diffusion coefficient for the case of the corrected immobile trajectories is as expected close to *D*_app_  ≈  0 *µ*m^2^ s^−1^ for all time points, thus providing a direct independent method for validating our data analysis approach as well as the resulting fit parameters.

A plot of the dependence of the apparent diffusion coefficient, *D*_app_, as a function of the characteristic correlation time *τ*_*D*_ for the STED-FCS data as in figure 4(b) and the error-corrected *D*_app_ (as given by equation (8) and from the fit that included both camera-blur and a localization error) as a function of time, *nt*_lag_, is shown in figure 5. This plot confirms that the STED-FCS data and the error-corrected SPT data is in very close agreement at short times, ≲2 ms, but that the SPT data is much more strongly confined at longer times. Providing further validation of our approach for correcting the raw SPT data for error resulting from camera blur and localization, we have also included the error-corrected data from the trajectories that were judged to be immobile as determined directly from the displacements. This shows as expected that the apparent diffusion coefficient for the case of the corrected immobile trajectories is close to *D*_app_  ≈  0 *µ*m^2^ s^−1^ for all time points. Finally, it is important to note that the only parameter that is required in order to superimpose the data as shown in figure 5 is an estimate of the localization error, Δ_*x*_,_*y*_. In this case the localization error was determined directly by fitting the raw data as described to mixed diffusion model 5 in table 1 with the localization error as a free parameter of the fit. The results in the case of the mobile data was Δ_*x*_,_*y*_  =  35.7  ±  0.5 nm while for the immobile data the best fit was Δ_*x*_,_*y*_  =  33.8  ±  0.6 nm. In contrast, the lower limit of the localization precision as determined from analysis of non-specifically absorbed QDs to a glass substrate that were imaged with similar conditions at the same set-up resulted in a lower limit of the localization precision of Δ_*x*_,_*y*_  ≈  28 nm [23]. This shows that already small errors in the estimation of Δ_*x*_,_*y*_ can already cause large differences in the determined values of *D*_*μ*_.

## 3. Conclusion

In this work, we have investigated the ten fold difference in the short-range diffusion coefficient of a phospholipid analogue in IA32 MEFs as determined by our STED-FCS measurements in comparison to that reported from high-speed SPT measurements. For this we performed a thorough examination of the experimental errors that are associated with each measurement technique. We subsequently applied our findings towards the analysis of the lateral mobility of a phospholipid analogue, biotin-cap-DPPE, labelled with sAv-QD655s that was acquired by SPT with a sampling frequency of  ≈1.8 kHz [23]. This examination shows that the analysis of high-speed SPT data is very susceptible to the effect of localization error. This effect of the localization error is most apparent upon converting the raw SPT data from the traditional MSD versus *t* space to the alternate MSD/(4*nt*_lag_) versus time format (equations (7) and (8)). This format can effectively also be thought of as a pseudo-*D*_app_ space that provides an accurate view of the actual *D*_app_ (*nt*_lag_) dependency at long times but that is dominated by measurement errors at very short times. In particular, we see that the plots in MSD/*t* versus *t* space diverges in the limit of *nt*_lag_  →  0 as a consequence of that the error contribution, Δ_*x*,*y*_/(*nt*_lag_), diverges. Consequently, we conclude that the contribution from the measurement errors is a key parameter in SPT analysis, and this error must be carefully accounted, in particular for analysis of data points that are acquired at very fast frame rates such that the limit of *nt*_lag_  →  0.

Our detailed analysis shows that our SPT data is best parametrized by use of a weighted least square fit to mixed diffusion model [15, 74] that has incorporated corrections for both camera blur and localization errors. This analysis resulted in a short-range diffusion coefficient *D*_*μ*_ of  ≈0.7–1.0 *µ*m^2^ s^−1^ for data analysis of time windows of 0.6  ⩽  *nt*_lag_  ⩽  *t*_end_ where *t*_end_ was 10, 25 or 50 ms. This result is in very good agreement with our previous STED-FCS data [24]. This analysis further results in comparable sizes for the confinement size *L* of  ≈110 nm for SPT and  ≈150 nm for STED-FCS but with a five fold greater confinement strength with the more invasive, very likely pauci-valent sAv-QD probes for SPT (*S*_Conf_  ≈  9–11) than for the less invasive Atto647N dye-probe for STED-FCS (*S*_Conf_  ≈  2). The convergence of the results for *D*_*μ*_ from the STED-FCS and SPT data suggests that the dependence on the diffusion measurements of the particular probe characteristics in this instance is minimal. This is despite that we used probes of very different characteristics. In the case of SPT, these were large (hydrodynamic radius of  ≈12 nm [70]) and pauci-valent (reported to contain 67  ±  10 biotin binding sites per QD [120] but where our labelling strategy combining brief labelling (1 min) followed by blocking with excess free biotin was validated to at least minimize probe-induced cross-linking [71]) sAv-QD probes bound to biotinylated DPPE phospholipids while in the case of the STED-FCS we used small, directly conjugated, mono-valent dye labelled DPPE phospholipids (figure 1). This result thus suggests that the short-range, intra-compartmental diffusion coefficient *D*_*μ*_ is consistent with the weaker dependence of the size of a membrane diffusing species on the 2D diffusion within a membrane as predicted by the Saffman–Delbrück relation [121]. On the other hand, the much slower long-range diffusion coefficient *D*_MACRO_ with SPT as compared to with STED-FCS signifies a much stronger dependence on the probe characteristics in this instance. This greater sensitivity of *D*_MACRO_ to the effects of using a more invasive larger probe is further consistent with earlier SPT data in cells that found that *D*_MACRO_ of a phospholipid that had been labelled with a gold nanoparticle was two fold smaller than similar measurements with a Cy3-labeled phospholipid analogue [6].

We further validated our analysis approach by a separate analysis of trajectories of molecules that were pre-determined to be immobile. This showed as expected that immobile molecules were characterized by a minimal short-range diffusion coefficient *D*_*μ*_  ≈  0.06 *µ*m^2^ s^−1^ and a long-range diffusion coefficient *D*_MACRO_  =  0 *µ*m^2^ s^−1^ thus resulting in an infinite confinement strength. The localization error, Δ_*x*_,_*y*_, was comparable for SPT data for mobile molecules (Δ_*x*_,_*y*_  =  35.7  ±  0.5 nm) and immobile molecules (Δ_*x*_,_*y*_  =  33.8  ±  0.6 nm). In contrast, analysis in a shorter time window of 0.6  ⩽  *nt*_lag_  ⩽  5 ms or alternatively for an unweighted fit resulted in faster short-range diffusion coefficients, *D*_*μ*_, but at the expense of that the corresponding analysis of data from immobile molecules also had similar larger short-range diffusion coefficients, *D*_*μ*_.

We have further shown a method for qualitative comparison of the SPT data, corrected for error measurement artefacts according to equation (12), and raw STED-FCS data (figure 5) which confirms the results of the quantitative comparison of this same data. A particular strength of this qualitative comparison is that it only requires one quantitative measure from the raw SPT data: the estimated localization error Δ_*x*_,_*y*_. In cases where the localization error can be determined precisely it is thus possible to perform this qualitative comparison independent of a particular diffusion model. This demonstration thus shows that experimental lateral diffusion measurements of a phospholipid analogue by STED-FCS and SPT can be shown to give similar qualitative and quantitative results if we also incorporate the recent understanding of the influence of localization noise and camera blur in the SPT analysis.

The very fast diffusion component that has been advocated from high-speed SPT measurements has in contrast been operationally defined by an unweighted fit to a Brownian diffusion model specifically to only data points 2  ⩽  *n*  ⩽  4 on a MSD versus *t* plot [75]. This analysis thus corresponds to time windows of 40(50)  ⩽  *n*  ⩽  80(100) *µ*s for data acquired at 50(40) kHz whereas the initial description of this analysis approach was for data acquired at 33 Hz thus corresponding to a time window of 66  ⩽  *n*  ⩽  122 ms [75]. This analysis is unfortunately not very detailed in the exact approach by which the experimental measurement errors were accounted for, in some cases referring only to comparison of a localization error of Δ_*x*_,_*y*_  ≈  17 nm as determined by analysing gold particles that were immobilized on poly-lysine coated coverslips and furthermore in a 10% acrylamide gel [6, 21]. Other measurements refer to control measurements at similar imaging conditions on cells by extrapolation of MSD versus *t* plots to zero time from linear fits of points 2  ⩽  *n*  ⩽  4 and by subtraction of the unspecified result from the remaining SPT data [7, 65]. In contrast, our analysis suggests that the exact determination of the localization error is a very important feature in SPT measurements, and in particular in the case of parametrization of a fast diffusion component in the limit where *nt*_lag_  →  0. We note in particular that an underestimation of the localization error at a specific time point, *nt*_lag_ increases the pseudo-apparent diffusion coefficient on an MSD versus t plot by the term $ \Delta _{x,y}^{2}$/(*nt*_lag_). For example, an underestimation of the precision by 10 nm corresponds to an error contribution of 0.2 *µ*m^2^ s^−1^ at *nt*_lag_  =  1 ms or 2 *µ*m^2^ s^−1^ at *nt*_lag_  =  50 *µ*s whereas an underestimation of the localization precision by 20 nm corresponds to an error contribution of 0.8 *µ*m^2^ s^−1^ at *nt*_lag_  =  1 ms or 8 *µ*m^2^ s^−1^ at *nt*_lag_  =  50 *µ*s.

We believe that resolving the ambiguity of the very different proposed diffusion coefficients of phospholipids in that the plasma membrane as determined by high-speed SPT measurements [6, 7, 21] in comparison to our recent STED-FCS measurements [24] is of fundamental biological importance. In the present work, we have shown that it is plausible that this discrepancy originates from the localization error in SPT measurements. Our results then suggest that the confinement strength in the plasma membrane is much weaker than what has been proposed from high-speed SPT measurements with gold probes. We hypothesize that such weaker confinement makes it more plausible that the cortical actin cytoskeleton plays a direct role in the efficiency of the initiation of various ligand-induced cell signalling events at the cell membrane that involve the specific oligomerization of signalling complexes, but that do not result in immobilization.
